# Virologic Outcomes With Lenacapavir in People With Human Immunodeficiency Virus: A Multicenter Real-World Study

**DOI:** 10.1093/ofid/ofag425

**Published:** 2026-07-09

**Authors:** Christopher Kaperak, Yijia Li, Beverly Sha, Monica Mercon, Karen Montes, Maria Sanes Guevara, Shivanjali Shankaran, Mariam Aziz, Catherine Creticos, Nancy Glick, Bijou R Hunt, Sharon Sam, Paul Djuricich, Andrew Merker, Aniruddha Hazra

**Affiliations:** Department of Medicine, Section of Infectious Diseases and Global Health, University of Chicago Medicine, Chicago, Illinois, USA; Department of Medicine, Division of Infectious Diseases, University of Pittsburgh, Pittsburgh, Pennsylvania, USA; Department of Internal Medicine, Division of Infectious Diseases, Rush University Medical Center, Chicago, Illinois, USA; Ruth M. Rothstein CORE Center, Cook County Health, Chicago, Illinois, USA; Ruth M. Rothstein CORE Center, Cook County Health, Chicago, Illinois, USA; Department of Medicine, Division of Infectious Diseases, University of Pittsburgh, Pittsburgh, Pennsylvania, USA; Department of Internal Medicine, Division of Infectious Diseases, Rush University Medical Center, Chicago, Illinois, USA; Department of Internal Medicine, Division of Infectious Diseases, Rush University Medical Center, Chicago, Illinois, USA; Howard Brown Health, Chicago, Illinois, USA; Sinai Infectious Disease Center, Sinai Chicago, Chicago, Illinois, USA; Sinai Infectious Disease Center, Sinai Chicago, Chicago, Illinois, USA; Sinai Infectious Disease Center, Sinai Chicago, Chicago, Illinois, USA; Department of Medicine, Section of Infectious Diseases and Global Health, University of Chicago Medicine, Chicago, Illinois, USA; Department of Medicine, Section of Infectious Diseases and Global Health, University of Chicago Medicine, Chicago, Illinois, USA; Department of Medicine, Section of Infectious Diseases and Global Health, University of Chicago Medicine, Chicago, Illinois, USA; Howard Brown Health, Chicago, Illinois, USA

**Keywords:** HIV, multidrug resistant HIV, lenacapavir, salvage anti-retroviral therapy, heavily treatment-experienced people with HIV

## Abstract

**Background:**

Lenacapavir (LEN), a first-in-class long-acting human immunodeficiency virus type 1 (HIV-1) capsid inhibitor, has demonstrated potent antiviral activity in heavily treatment-experienced (HTE) people with HIV (PWH) in clinical trials, but real-world data remain limited. We describe outcomes from 6 US HIV clinics using LEN-based regimens in a largely HTE cohort of PWH to assess virologic response, regimen potency, and tolerability.

**Methods:**

We conducted a multicenter retrospective cohort study of adults with HIV who initiated LEN between November 2020 and June 2025 at 6 clinics in Chicago, Illinois and Pittsburgh, Pennsylvania. Demographic, clinical, and genotypic data were abstracted from electronic health records. Virologic suppression was defined as HIV viral load <200 copies/mL. Stanford genotypic susceptibility scores (S-GSS) were calculated to assess regimen potency. Wilcoxon signed-rank tests compared antiretroviral therapy pill burden and regimen potency before and after LEN initiation.

**Results:**

Seventy PWH initiated LEN. Median follow-up was 12 months. At baseline, 18 (26%) PWH with available genotypes had multidrug-resistant HIV, and 54% had unsuppressed virus. Among 38 PWH with unsuppressed virus, 34 (89%) achieved viral suppression, and none of the PWH with suppressed HIV experienced rebound. LEN significantly improved regimen potency (*P* < .00005) and reduced daily pill burden from 2 tablets to 1 tablet (*P* < .00005). Injection site reactions occurred in 30% and led to discontinuation in 1 person.

**Conclusions:**

In this real-world cohort, LEN-based regimens achieved high rates of virologic suppression, improved regimen activity, and reduced pill burden with good tolerability. LEN represents a promising salvage therapy for PWH, warranting equitable and implementation-focused integration into HIV care.

## Teaser

Lenacapavir-based regimens achieved high rates of virologic suppression and reduced pill burden among people with HIV across 6 US clinics, demonstrating strong real-world effectiveness and tolerability and highlighting its role as a key long-acting salvage therapy.

Lenacapavir (LEN), a long-acting human immunodeficiency virus (HIV) capsid inhibitor, was approved for use in heavily treatment-experienced (HTE) people with HIV type 1 (HIV-1) (PWH) in August 2022 in the European Union [1] and in the United States in December 2022 [2]. These approvals were largely based on the results of the CAPELLA trial, a phase 3 clinical trial that enrolled 72 HTE PWH with multidrug-resistant (MDR) HIV-1 infection, and found significant viral load (VL) decrease when used as functional monotherapy and high rates of virologic suppression when used with a functional optimized background regimen (OBR) when given at 6-month intervals subcutaneously [3]. As a first-in-class medication, LEN has also been the subject of trials investigating its use in early HIV infection without previous resistance [4], as a switch strategy co-formulated with bictegravir [5] or islatravir [6] for people with virologic suppression, and has also been approved in the United States and endorsed globally as a twice-yearly subcutaneous injection for use as HIV preexposure prophylaxis (PrEP) based on the results of its 2 registrational trials, PURPOSE1 and PURPOSE2 [7–10].

Additional data beyond the initial CAPELLA trial findings are limited. From CAPELLA trial extension data through 104 weeks, 63% (45) of participants were confirmed to have an HIV VL <200 copies/mL and 75% (54) of participants were reported to have 1 or more medication-related injection site reactions (ISRs), with only 1 stopping the medication due this issue [11]. The Observational Pharmaco-Epidemiology Research & Analysis (OPERA) cohort identified 116 PWH receiving LEN, with 92% probability of maintaining a VL of <200 copies/mL, 76% probability of becoming newly suppressed to VL <200 copies/mL, and 86% adherence to on-time injections; genotypes were not available due the nature of their data collection [12]. Saberi et al published a summary of National Clinician Consultation Center cases of transitioning antiretroviral therapy (ART) regimens to LEN plus cabotegravir (CAB) with or without rilpivirine, typically in cases of difficulty with adherence to oral ART or ongoing viremia. Although follow-up data were limited, HIV VLs decreased among those with available data [13]. We previously reported our own HIV clinic's experience with 10 PWH receiving LEN, where 7 of 9 with available follow-up data remained suppressed for a median of 320 days [14]. However, more data regarding HIV VLs, CD4 count trends, and tolerability outside of clinical trial settings are needed. Here, we describe 6 US HIV clinics’ early real-world experience with twice-yearly subcutaneous LEN for HTE PWH, along with impacts on ART pill burden and activity of patients’ ART regimen. Our study describes LEN use in people with MDR HIV as defined by Stanford genotypic susceptibility score (S-GSS), OBR adherence data, and viral suppression to <50 copies/mL.

## METHODS

We conducted a multicenter retrospective cohort study of HTE PWH who started LEN from 6 HIV clinics: University of Chicago (UC), University of Pittsburgh Medical Center (UPMC), CORE Center (CC), Rush University Medical Center (RUMC), Howard Brown Health (HBH), and Sinai Chicago (SC). Data were collected from 13 November 2020 to 4 April 2025, at UC, UPMC, RUMC, HBH, and SC. Two PWH in this cohort started LEN prior to US Food and Drug Administration approval. One from HBH was part of the CAPELLA trial and continued LEN after trial completion. The other was from RUMC and received LEN via compassionate use. Due to different timing in data availability from CC, data regarding LEN initiation were collected from CC through 26 June 2025. Local institutional review board approval for series inclusion was obtained and maintained in accordance with standards and requirements at each site. De-identified data were securely transferred, stored, and analyzed at the coordinating site. Clinics were selected based on location either in or near Chicago and interest of representatives from each institution. Clinics contacted that had no PWH receiving LEN were excluded. At all institutions, participants were included if they were at least 18 years old and had received at least 1 dose of LEN. Data was collected locally by review of each institution's respective electronic medical record. Demographic data was collected, including age at LEN initiation, sex assigned at birth, gender identity, race, ethnicity, zip code, insurance type, history of substance use disorder, active substance use, history of injection drug use, and housing status at time of LEN initiation. Clinical characteristics were collected, including year of HIV diagnosis, body mass index (BMI) at start of LEN, previous documented ART regimens (including specification of which ART regimen a participant was prescribed immediately preceding LEN initiation), reason for LEN initiation, dates of all LEN injections, OBR, adherence to OBR, whether a participant stopped LEN, ISRs, hepatitis B/C screening, HIV subtype, HIV RNA and archival DNA genotypes, coreceptor tropism status, HIV VL before and after starting LEN, and CD4^+^ T-cell count before and after starting LEN. Previous and current ART regimens were used to identify PWH who were defined as HTE by using criteria described by Hsu et al in analyzing the OPERA cohort, although previous or current use of fostemsavir or ibalizumab were added to their criteria [15].

Stanford genetic susceptibility rating scores (S-GSR) [16, 17] were used to calculate total S-GSS values to determine how many active agents PWH had in their regimen among nucleoside reverse transcriptase inhibitor (NRTI), nonnucleoside reverse transcriptase inhibitor (NNRTI), protease inhibitor (PI), and integrase strand transfer inhibitor (INSTI) classes [17]. Per the Stanford University HIV Drug Resistance Database (HIVdb), each ART agent receives a total penalty score based on HIV genotypic mutations. A score of ≥60 equates to an S-GSR of 0; 30 to <60 to an S-GSR of 0.25; 15 to <30 to an S-GSR of 0.5; and 10 to <15 to an S-GSR of 0.75. A drug total penalty score of <10 equates to an S-GSR of 1 [16, 17]. An S-GSR score of 0.25 or lower (total penalty score of 30 or higher) was used to classify an individual's HIV genotype as resistant to a particular agent for the purposes of identifying MDR HIV. MDR HIV was defined using similar criteria for inclusion in CAPELLA: genotypic resistance to at least 2 ART agents from at least 3 out of 4 of the main ART classes (NRTI, NNRTI, PI, and INSTI) without using M184V/I to assess emtricitabine and lamivudine resistance. Additionally, there could be no more than 2 fully active ART agents from the main 4 classes able to be combined effectively (eg, some PWH were excluded due to darunavir, doravirine, and tenofovir all remaining active despite otherwise broad resistance) [3]. S-GSS values were calculated centrally by 3 of the authors to minimize variation in responses based on provided genotypes. Fostemsavir, maraviroc, and ibalizumab, when present, were assumed to have a value of 1. LEN was similarly assumed to have a value of 1 upon initiation, as resistance to LEN is uncommon, especially in HIV subtype B, the most prevalent subtype in North America [18]. Viral fitness loss from accumulated mutations was not included in the calculation of S-GSS values. Regimen potency was defined using these S-GSS values.

Descriptive statistics were used to summarize demographic and clinical data, both before and after LEN initiation. Survival analysis methods were used to summarize time to HIV VL suppression to <200 copies/mL as a primary endpoint and 50 copies/mL as a secondary endpoint among PWH who were not suppressed prior to starting LEN. Log-rank tests were used to compare time to suppression among people with and without MDR HIV; if the proportional hazards assumption was violated, only observations prior were compared. Wilcoxon signed-rank tests were used to compare the number of ART-containing pills taken per day, the number of ART agents PWH were exposed to, and the number of active agents based on S-GSS values in the regimens PWH received before LEN and with LEN, again divided by MDR HIV status. Injectable and intravenous regimens did not count toward pill count totals, and pills dosed multiple times per day, such as twice-daily dolutegravir, counted for each dose given. Cox proportional hazard methods were used to analyze associations with new VL reduction to <50 copies/mL. Figures and statistical analysis were completed using Stata Statistical Software, Release 19 (StataCorp LLC, College Station, TX, USA).

## RESULTS

### Baseline Data

Among the 6 clinics, 70 PWH initiated LEN (Table 1). Among this cohort, 18 (26%) had MDR HIV based on available genotypes. Cohort demographics are notable for 38 (54%) identifying as cisgender men, 51 (73%) identifying as Black, and 60 (86%) identifying as non-Hispanic. Fifty-one (73%) received either Medicaid or Medicare insurance. Self-reported substance use history (which included marijuana and heavy alcohol use) was present in 26 (37%) PWH. Seven (10%) reported unstable housing. None of the above characteristics differed by the PWH's MDR HIV status. The average age when starting LEN was 52.5 (standard deviation [SD], 12.5) years for the entire cohort, although people with MDR HIV were older on average (57.6 [SD, 13.0] years vs 50.8 [SD, 11.9] years; *P* = .043). The average time since HIV diagnosis was 24.0 (SD, 9.5) years in the 67 PWH with available data, and was also higher among people with MDR HIV (28.3 [SD, 6.0] years vs 22.6 [SD, 10.0] years; *P* = .031). Using a VL cutoff of 200 copies/mL, 54% of the cohort (38 PWH) had unsuppressed virus when starting LEN, 4 of whom had a VL of >100 000 copies/mL (Table 2). HIV VL characteristics did not differ by people's MDR HIV status (Table 1). The cohort's median CD4^+^ T-cell count was 271 cells/μL prior to LEN start. People with MDR HIV had a higher median CD4^+^ T-cell count (461.5 vs 210.5 cells/μL, *P* = .022). Average BMI at time of LEN initiation was 27.5 (SD, 8.3) kg/m^2^. Besides having MDR HIV (per unstandardized respondent report), other commonly reported reasons to start LEN were simplification for PWH already suppressed to <200 copies/mL and virologic failure, each present for 29 (41% of PWH). Participating sites could also comment with additional reasons, for which 28 (40%) had additional reasons, with intolerance to oral ART (nausea or dysphagia) and housing instability being the 2 most common additional reasons reported for starting LEN. PWH without MDR HIV made up the majority of “Other” reasons for starting LEN, with “Other” being reported for 26 (50%) PWH in that group. Fifty-three (76%) PWH qualified as HTE using the modified OPERA definition, with all people with MDR HIV being HTE.

**Table 1.** Cohort Characteristics

Characteristic	Overall	MDR HIV	Non-MDR HIV	*P* Value
No. of participants	70	18	52	
Age when starting LEN, y, mean (SD)	52.5 (12.5)	57.6 (13.0)	50.8 (11.9)	.043
Sex assigned at birth				.50
Female	32 (46%)	7 (39%)	25 (48%)	
Male	38 (54%)	11 (61%)	27 (52%)	
Gender				.50
Cisgender man	38 (54%)	11 (61%)	27 (52%)	
Cisgender woman	32 (46%)	7 (39%)	25 (48%)	
Race				.53
Black or African American	51 (73%)	12 (67%)	39 (75%)	
More than 1 race	1 (1%)	0 (0%)	1 (2%)	
Unknown/not reported	3 (4%)	0 (0%)	3 (6%)	
White	15 (21%)	6 (33%)	9 (17%)	
Ethnicity				.43
Hispanic or Latino	10 (14%)	1 (6%)	9 (17%)	
Not Hispanic or Latino	60 (86%)	17 (94%)	43 (83%)	
Insurance plan at time of LEN initiation				.055
Medicaid	30 (43%)	6 (33%)	24 (46%)	
Medicare	21 (30%)	10 (56%)	11 (21%)	
Private insurance	16 (23%)	2 (11%)	14 (27%)	
Self-pay	3 (4%)	0 (0%)	3 (6%)	
History of substance use disorder				.46
No	44 (63%)	10 (56%)	34 (65%)	
Yes	26 (37%)	8 (44%)	18 (35%)	
Housing status at time of LEN initiation				.31
Stably housed	60 (86%)	17 (94%)	43 (83%)	
Unknown	3 (4%)	1 (6%)	2 (4%)	
Unstably housed/unhoused	7 (10%)	0 (0%)	7 (13%)	
BMI at LEN start, kg/m^2^, mean (SD)	27.5 (8.3) (n = 67)	27.9 (8.2) (n = 18)	27.4 (8.4) (n = 49)	.83
Number of years living with HIV before starting LEN, mean (SD)	24.0 (9.5)	28.3 (6.0) (n = 17)	22.6 (10.0) (n = 50)	.031
Baseline HIV VL, copies/mL, median (range)	391 (0–758 000)	139.5 (0–758 000)	533.5 (0–59 000)	.64
Virally suppressed <200 copies/mL at LEN start				.33
Yes	32 (46%)	10 (56%)	22 (42%)	
No	38 (54%)	8 (44%)	30 (58%)	
Virally suppressed <50 copies/mL at LEN start				.37
Yes	25 (36%)	8 (44%)	17 (33%)	
No	45 (64%)	10 (56%)	35 (67%)	
Patients with VL >10 000 copies/mL				.72
Yes	21 (30%)	6 (33%)	15 (29%)	
No	49 (70%)	12 (67%)	37 (71%)	
Patients with VL >100 000 copies/mL				1.00
Yes	4 (6%)	1 (6%)	3 (6%)	
No	66 (94%)	17 (94%)	49 (94%)	
Last CD4 count before LEN started, cells/μL, median (range)	271 (0–1923)	461.5 (20–1177)	210.5 (0–1923)	.022
Reason for LEN initiation				
Simplification/convenience of regimen (VL already <200 copies/mL)	29 (41%)	10 (56%)	19 (37%)	.16
Virologic failure of current regimen	29 (41%)	7 (39%)	22 (42%)	.80
MDR HIV (as defined by respondents)	45 (64%)	15 (83%)	30 (58%)	.05
Drug–drug interaction in pre-LEN regimen	2 (3%)	1 (6%)	1 (2%)	.45
Other (oral intolerance, housing instability)	28 (40%)	2 (11%)	26 (50%)	.004
NRTI resistance^a^	55 (81%)	18 (100%)	37 (74%)	.015
NNRTI resistance^a^	51 (75%)	18 (100%)	33 (66%)	.003
PI resistance^a^	16 (24%)	11 (61%)	5 (10%)	<.001
INSTI resistance^a^	28 (41%)	11 (61%)	17 (34%)	.045
Heavily treatment experienced				.004
Yes	53 (76%)	18 (100%)	35 (67%)	
No	17 (24%)	0 (0%)	17 (33%)	
S-GSS, median (range)^a^	2 (0–3.25)	1.5 (0.25–2.75)	2.25 (0–3.25)	.002
Estimated number of active agents pre-LEN regimen^a^				.001
Less than 1	8 (11%)	4 (22%)	4 (8%)	
At least 1	17 (24%)	9 (50%)	8 (15%)	
At least 2	43 (61%)	5 (28%)	38 (73%)	
Unknown	2 (3%)	0 (0%)	2 (4%)	

Data are presented as No. (%) unless otherwise indicated. Variables with missing data identified as either “unknown” or, when presented as mean (SD), with the number of participants with available data.

Abbreviations: BMI, body mass index; HIV, human immunodeficiency virus; INSTI, integrase strand transfer inhibitor; LEN, lenacapavir; MDR, multidrug resistant; NNRTI, nonnucleoside reverse transcriptase inhibitor; NRTI, nucleoside reverse transcriptase inhibitor; PI, protease inhibitor; SD, standard deviation; S-GSS, Stanford genotypic susceptibility score; VL, viral load.

^a^Two participants excluded due to no available HIV-1 genotype results.

**Table 2.** Cohort With HIV Viral Load >200 Copies/mL Pre-Lenacapavir Regimen (n = 38)

Characteristic	Overall	MDR HIV	Non-MDR HIV	*P* Value
No. of participants	38	8	30	
Last HIV VL before LEN start, copies/mL, median (range)	15 627 (234–758 000)	29 296 (415–758 000)	9801 (234–590 000)	.28
Patients with VL >100 000 copies/mL				1.00
Yes	4 (11%)	1 (12%)	3 (10%)	
No	34 (89%)	7 (88%)	27 (90%)	
Last CD4 count before LEN start, cells/μL, median (range)	157 (0–1177)	200 (20–1177)	149 (0–906)	.22
Resistance to 2 or more agents (n = 37)^a^				
NRTI	30 (81%)	8 (100%)	22 (76%)	.31
NNRTI	27 (73%)	8 (100%)	19 (66%)	.079
PI	6 (16%)	3 (38%)	3 (10%)	.10
INSTI	18 (49%)	8 (100%)	10 (34%)	.001
Heavily treatment experienced				.082
Yes	28 (74%)	8 (100%)	20 (67%)	
No	10 (26%)	0 (0%)	10 (33%)	
S-GSS, median (range)	2 (0–3)	1.125 (0.25–2.25)	2 (0–3)	.017
Estimated number of active agents pre-LEN regimen per S-GSS				.003
Less than 1	7 (18%)	3 (38%)	4 (13%)	
At least 1	7 (18%)	4 (50%)	3 (10%)	
At least 2	23 (61%)	1 (12%)	22 (73%)	
Unknown	1 (3%)	0 (0%)	1 (3%)	

Data are presented as No. (%) unless otherwise indicated.

Abbreviations: HIV, human immunodeficiency virus; INSTI, integrase strand transfer inhibitor; LEN, lenacapavir; MDR, multidrug resistant; NNRTI, nonnucleoside reverse transcriptase inhibitor; NRTI, nucleoside reverse transcriptase inhibitor; PI, protease inhibitor; S-GSS, Stanford genotypic susceptibility score; VL, viral load.

^a^One participant excluded due to no available HIV-1 genotype results.

### Genotypic Data

HIV genotype data was available for 68 of the 70 PWH in the cohort, which included archival DNA genotypes in 14 PWH (Tables 1 and 2). Frequencies of individual mutations were compiled from available genotypes (Supplementary Tables 1–3). The majority of PWH had HIV resistant to 2 or more agents in the NRTI (55 [81%]) and NNRTI (51 [75%]) drug classes, including all people with MDR HIV. HIV with INSTI resistance was present in 28 (41%) and PI resistance was present in 16 (24%) cases of HIV. Resistance to the 4 major ART classes was more common among people with MDR HIV when assessing the entire cohort, although that relationship was only seen among the INSTI class in PWH with viremia (*P* = .001). Using S-GSS estimation, only 43 (61%) of PWH had at least 2 active agents in their prescribed ART regimen before starting LEN, with 8 (11%) having the equivalent of <1 active agent. The median number of active agents per S-GSS was lower in the group with MDR HIV (S-GSS of 1.5 vs 2.25, *P* = .002). See Supplementary Tables 1–3 for more detailed genotypic information, including major versus other mutations as defined by the Stanford HIVdb [16].

### Results After Starting Lenacapavir

**Figure 1.**
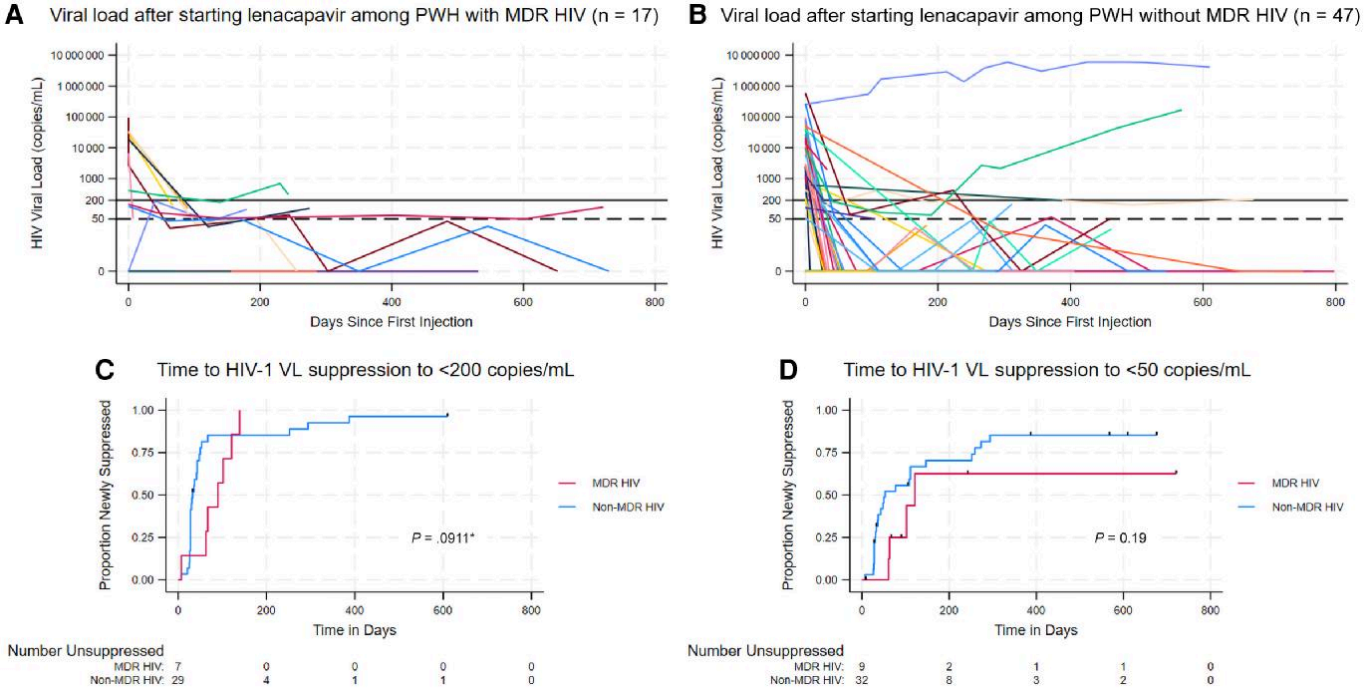
Viral load (VL) trajectory after lenacapavir (LEN) initiation. The x-axis is cut off at 800 days; 2 people with HIV (PWH) were on LEN for >800 days. All of their VLs were <200 copies/mL. *A*, Plot shows HIV-1 VL in a logarithmic base 10 scale among people with multidrug-resistant (MDR) HIV. VL at day zero was their VL collected either on the day of starting LEN or the closest available level prior to starting. One PWH in the cohort is excluded as they did not have an available VL. *B*, Plot shows 47 people without MDR HIV using the same specifications as in (*A*). Five PWH are excluded due to lack of follow-up data. *C*, Plot shows time to new HIV-1 VL suppression among 36 PWH, defined using 200 copies/mL. Black tick marks show censored PWH, split by MDR HIV status. Censoring occurred for never being suppressed at most recent follow-up, whether or not they were still prescribed LEN. Two PWH are excluded from VL <200 copies/mL for lack of follow-up data. Log-rank test was completed, although it was only used in comparing the first 100 days of follow-up time due to the assumption of proportional hazards becoming violated a second time after that time point, which is denoted by an asterisk as Figure C should be interpreted with caution. *D*, Plot shows time to new HIV VL suppression among 41 PWH, defined using a cutoff of <50 copies/mL. The same specifications are used. Five PWH are excluded due to lack of follow-up VL after starting LEN. Log-rank test completed, not statistically significant.

After starting LEN, INSTI was the most common ART class, present in 70% of the PWH's OBR (Table 3). The NRTI, NNRTI, and PI classes were all present in PWH's regimens, along with ibalizumab and fostemsavir as other salvage therapies. No PWH remained on maraviroc, and no PWH were prescribed enfuvirtide in their immediate pre-LEN or post-LEN regimens. As of most recent available follow-up, 62 (89%) PWH remained on LEN, with no difference based on a PWH's MDR HIV status. Median days of confirmed active LEN is 377 days among the entire cohort, and 387 days among the 62 PWH who remain on LEN, with no difference identified based on PWH's MDR status. Follow-up HIV VL data were available for 64 PWH (Figure 1*A* and 1*B*). Of the PWH who are no longer prescribed LEN, 4 were lost to follow-up and 2 died while receiving LEN (HIV VL <200 copies/mL for both PWH). One self-discontinued due to ISRs and 1 was physician-guided based on poor OBR adherence. ISRs were common, confirmed in at least 30% of the cohort. Self-reported adherence to OBR was available in 65 (93%) of PWH per review of notes, with 55 (79%) reporting missing <1 dose of their OBR per week and 6 (9%) reporting missing ≥3 doses per week. No differences were identified in these factors based on MDR HIV status.

**Table 3.** Outcomes Post–Lenacapavir Initiation (N = 70)

Outcome	Overall	MDR HIV	Non-MDR HIV	*P* Value
No. of participants	70	18	52	
Medications in OBR				
NRTI	35 (50%)	11 (61%)	24 (46%)	.27
NNRTI	22 (31%)	3 (17%)	19 (37%)	.12
PI	17 (24%)	6 (33%)	11 (21%)	.35
INSTI	49 (70%)	11 (61%)	38 (73%)	.34
Ibalizumab	7 (10%)	1 (6%)	6 (12%)	.42
Fostemsavir	8 (11%)	5 (28%)	3 (6%)	.023
Still on LEN				.67
Yes	62 (89%)	17 (94%)	45 (87%)	
No	8 (11%)	1 (6%)	7 (13%)	
HIV VL newly <200 copies/mL on LEN (n = 38)				.57
Yes	31 (82%)	6 (75%)	25 (83%)	
No	5 (13%)	1 (12.5%)	4 (13%)	
Unknown	2 (5%)	1 (12.5%)	1 (3%)	
Latest CD4 count since LEN start, cells/μL, median (range)	297 (2.5–1064) (n = 52)	495 (20–1064) (n = 11)	259 (2.5–1048) (n = 41)	.031
Days observed on LEN, median (range)				
Entire cohort	377 (8–1593)	269 (22–1228)	396 (8–1593)	.60
PWH with active LEN	387 (8–1593) (n = 62)	309 (22–1228) (n = 17)	397 (8–1593) (n = 45)	.69
Injection site reaction				.76
Minor	19 (27%)	6 (33%)	13 (25%)	
Moderate (needed medical visit)	2 (3%)	0 (0%)	2 (4%)	
None reported	49 (70%)	12 (67%)	37 (71%)	
Self-reported adherence to OBR				.77
Good (<1 missed dose per week)	55 (79%)	14 (78%)	41 (79%)	
Fair (1 missed dose per week)	1 (1%)	0 (0%)	1 (2%)	
Modest (2 missed doses/week)	3 (4%)	0 (0%)	3 (6%)	
Poor (≥3 missed doses/week)	6 (9%)	2 (11%)	4 (8%)	
Unknown	5 (7%)	2 (11%)	3 (6%)	
S-GSS, median (range)	2.5 (2–4)	2.375 (2–3.75)	2.875 (2–4)	.67
Estimated number of active agents in OBR				.255
At least 1	38 (54%)	13 (72%)	25 (48%)	
At least 2	30 (43%)	5 (28%)	25 (48%)	
Unknown	2 (3%)	0 (0%)	2 (4%)	
Wilcoxon signed-rank test results	Overall	MDR HIV	Non-MDR HIV	
S-GSS scores before LEN vs with LEN (n = 68)	*P* < .00005	*P* = .0002	*P* = .0001	
Number of agents pre-LEN vs with LEN	*P* = .91	*P* = 1.0	*P* = .89	
Daily pill burden pre-LEN vs with LEN	*P* < .00005	*P* = .0044	*P* < .00005	

Data are presented as No. (%) or median (IQR). Variables with missing data identified as either “unknown” or, when presented as median (IQR), with the number of participants with available data.

Abbreviations: HIV, human immunodeficiency virus; INSTI, integrase strand transfer inhibitor; IQR, interquartile range; LEN, lenacapavir; MDR, multidrug resistant; NNRTI, nonnucleoside reverse transcriptase inhibitor; NRTI, nucleoside reverse transcriptase inhibitor; OBR, optimized background regimen; PI, protease inhibitor; PWH, people with human immunodeficiency virus; S-GSS, Stanford genotypic susceptibility score; VL, viral load.

Among the 32 PWH with suppressed HIV (VL <200 copies/mL) at baseline, there were no identified virologic failures. Of the 38 PWH without suppressed HIV prior to starting LEN, 34 of them achieved HIV suppression at least once (defined as a VL <200 copies/mL one time) (Figure 1*C* and 1*D*), and 31 (82%) have continued to have fully suppressed HIV since starting LEN, with no significant difference based on MDR HIV status. No differences based on MDR HIV status were identified in time to VL suppression to <200 or <50 copies/mL using log-rank tests, although the comparison to new suppression to <200 copies/mL violates the proportional hazards assumption (Figure 1*C* and 1*D*). Of the 7 PWH without confirmed HIV suppression, 2 did not have an available HIV VL. Two PWH, 1 with MDR HIV and 1 without MDR HIV, had HIV VLs <1000 copies/mL, self-reported excellent OBR adherence, and remained on LEN at the time of data cutoff. One PWH without MDR HIV demonstrated a 0.85 log reduction in HIV VL after 56 days in the setting of dysphagia and missing about 2 days of their OBR per week, with subsequent follow-up pending. One PWH without MDR HIV suppressed to <200 copies/mL but who stopped LEN voluntarily due to ISRs then had poor adherence to their OBR. One PWH with significant dysphagia and without MDR HIV self-discontinued their OBR while receiving LEN and developed presumed resistance.

**Figure 2.**
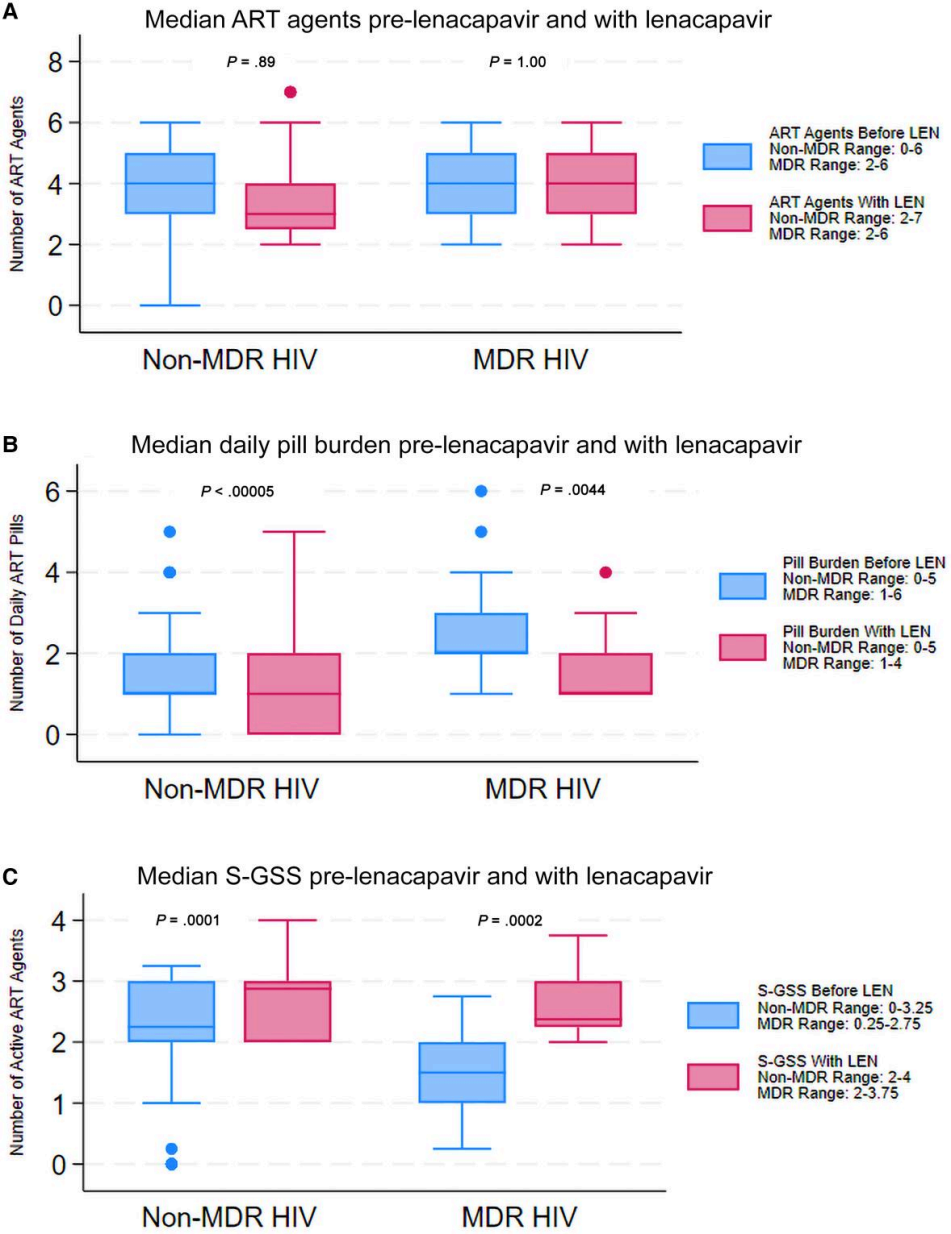
Number of antiretroviral therapy (ART) agents, pill burden, and Stanford genotypic susceptibility score (S-GSS) before vs with lenacapavir (LEN), compared using Wilcoxon signed-rank testing and divided by multidrug-resistant (MDR) HIV status (n = 18 for group with MDR HIV, n = 52 for group without MDR HIV). *A*, Plot compares the median number of antiretroviral agents included in the entire cohort's regimens before starting LEN and with LEN. Boosting agents, such as cobicistat, and nondaily medications, such as ibalizumab and injectable cabotegravir/rilpivirine, were included. No difference in median antiretroviral agent exposure on a daily basis was identified in either group. *B*, Plot compares the median number of antiretroviral pills in the cohort's regimens prior to including LEN and with LEN. Nonoral medications were excluded, and medications taken multiple times per day, such as twice-daily dolutegravir, counted for each pill taken. This cohort had a reduction of about 1 pill per day in their daily ART regimen in both groups. *C*, Plot compares the median S-GSS in the members of the cohort with available HIV genotype data (RNA or DNA) before starting LEN and with LEN (n = 50 for group without MDR HIV). LEN was assumed to be fully active as it was a new exposure for all PWH. This cohort had an increase in median S-GSS by about 0.5 points; notably, people with MDR HIV had an increase in median S-GSS of nearly 1 agent, increasing from 1.5 to 2.375 with creation of a LEN-containing regimen. No PWH had a LEN-containing regimen with <2 active agents.

LEN-based regimens varied greatly based on clinic, provider, and individual PWH's needs in terms of whether LEN was added on to a regimen, if LEN replaced an agent, if several agents with different routes of administration were used or changed, or if regimens were converted to an injectable-only regimen. With the addition of LEN into PWH's regimens, we did not find a difference in the number of daily ART agents PWH were exposed to (*P* = .91), including when stratified by MDR HIV (*P* = 1.0 among people with MDR HIV; *P* = .89 without MDR HIV) (Figure 2*A*). PWH received a median of 4 ART agents (interquartile range [IQR], 3–5) in both the pre-LEN and LEN-containing regimens (Supplementary Table 4). This calculation included boosting agents, such as cobicistat, and long-acting agents, such as intramuscular CAB, toward the number of daily agents. In contrast, the number of pills needed in PWH's ART regimens before starting LEN and in the LEN-containing regimens significantly decreased for the whole cohort (*P* < .00005) and when stratifying by presence of MDR HIV (*P* = .0044 among people with MDR HIV, *P* < .00005 among people without). There were 2 pills (IQR, 1–2) in the pre-LEN regimens and 1 pill (IQR, 0–2) in the LEN-containing regimens (Figure 2*B*). Twenty-one PWH had non-oral ART regimens, all of them with intramuscular CAB and/or intramuscular rilpivirine, with all of them having non-MDR HIV. Three of these PWH required ibalizumab infusions in addition to injectable ART (Supplementary Table 5).

Updated S-GSS values were calculated in the 68 PWH with pre-LEN HIV genotypes available, based on their new LEN-based regimens (Table 3). Genotypes collected after LEN initiation were not used as adherence varied among PWH. With LEN, 30 (43%) of PWH had at least 2 active OBR agents, and the other 38 (54%) had >1 confirmed active OBR agent. No PWH had <1 active agent in their OBR. Using Wilcoxon signed-rank testing, the median S-GSS (inclusive of LEN) increased from 2.0 to 2.5 with starting LEN (*P* < .00005) (Table 3), with similar results among people with and without MDR HIV (Table 3, Figure 2*C*).

In a secondary analysis, 25 (36%) PWH had HIV suppressed to <50 copies/mL at baseline (Table 1). Of these, 1 PWH experienced HIV VL increase to 188 copies/mL after 40 days and has continued to have HIV VL between 50 and 200 copies/mL through 180 days on LEN. Twenty-two PWH who started LEN at an HIV VL <50 copies/mL stayed at <50 copies/mL, with the other 2 PWH still due for a subsequent VL measurement. Forty-five PWH (64%) started LEN with an HIV VL >50 copies/mL. Of those, 41 had available follow-up data. Thirty-two (78%) PWH achieved new HIV suppression to ≤50 copies/mL, while the other 9 PWH (22%) continued to have HIV VL >50 copies/mL (Figure 1*C* and 1*D*). In total, 54 (77%) of PWH were confirmed to have either maintained or achieved new VL suppression to <50 copies/mL.

We evaluated factors associated with new VL suppression to <50 copies/mL in PWH. Four of 45 were excluded due to absence of follow-up data. The S-GSS of the LEN-containing regimen was associated with a lower likelihood of achieving new suppression to <50 copies/mL (hazard ratio [HR], 0.35 [95% confidence interval {CI}, .17–.72]). No significant associations were identified in univariate analyses of MDR HIV status, HTE status, S-GSS of the pre-LEN regimens, baseline CD4 count, and OBR adherence. In the multivariate analysis including these variables, the adjusted HR of new suppression associated with the S-GSS of the LEN-containing regimen was 0.19 (95% CI, .072–.48; *P* = .001) and the HR of new suppression associated with the S-GSS of the pre-LEN regimen was 1.73 (95% CI, 1.03–2.88; *P* = .037). No significant association with MDR HIV or HTE status, baseline CD4, and OBR adherence was found with new VL suppression to <50 copies/mL (Table 4).

**Table 4.** Cox Proportional Hazards Analysis: New Suppression to <50 Copies/mL (n = 41)

Covariate	Unadjusted HR (95% CI)	*P* Value	Adjusted HR (95% CI)	*P* Value
S-GSS of pre-LEN regimen (n = 39)	1.18 (.79–1.8)	.42	1.73 (1.03–2.88)	.037
S- GSS of LEN-containing regimen (n = 39)	0.35 (.17–.72)	.004	0.19 (.072–.48)	.001
Baseline CD4 count >200 cells/μL or not	0.88 (.42–1.9)	.74	1.12 (.48–2.64)	.79
Adherence to OBR: <1 dose per week or not	1.11 (.47–2.6)	.81	0.51 (.18–1.5)	.214
Multidrug-resistant HIV	0.50 (.17–1.4)	.20	0.89 (.26–3.0)	.85
Heavily treatment experienced	0.69 (.30–1.6)	.38	0.58 (.23–1.5)	.26

Forty-one participants were included in analysis as 4 did not have follow-up viral load testing available. Adjusted HR analysis included all 6 covariates.

Abbreviations: CI, confidence interval; HIV, human immunodeficiency virus; HR, hazard ratio; LEN, lenacapavir; OBR, optimized background regimen; S-GSS, Stanford genotypic susceptibility score.

## DISCUSSION

In this multicenter, real-world cohort of mostly HTE PWH, injectable LEN–based regimens achieved high rates of virologic suppression among people with and without MDR HIV, demonstrating clinical effectiveness comparable to its pivotal clinical trials and early observational studies. These findings reinforce LEN's role as a potent and durable option in the management of MDR HIV and among people with other barriers to adherence, particularly when combined with an OBR containing at least 1 fully active agent.

Among PWH with unsuppressed virus at baseline, >80% achieved virologic suppression to <200 copies/mL, >70% achieved suppression to <50 copies/mL, and none of the PWH who were already suppressed experienced virologic rebound to >200 copies/mL during follow-up, with no significant difference based on having a history of MDR HIV. These results are consistent with CAPELLA's open-label extension, in which 61% of participants maintained VLs <50 copies/mL at week 156 as well as with the 73% probability of sustained suppression observed in the OPERA cohort and the large declines in absolute VL seen in National Clinician Consultation Center data [12, 13, 19]. The alignment of outcomes across clinical trials and real-world settings underscores LEN's robustness and reproducibility even among individuals with extensive resistance profiles and prior treatment failures.

Notably, LEN initiation led to significant improvement in regimen potency as measured by S-GSS, underscoring its ability to strengthen salvage regimens even in the presence of extensive resistance across traditional drug classes. This unique feature makes LEN an important therapeutic anchor in complex treatment regimens. While the model suggested that higher postswitch S-GSS was associated with a lower likelihood of achieving VL <50 copies/mL, examination of individual-level data did not reveal a consistent resistance-related pattern; rather, outcomes appeared to be driven by a small number of outliers who had difficulty with OBR adherence, including all 3 PWH summarized above who did not achieve VL suppression <1000 copies/mL. Many individuals with lower S-GSS values achieved suppression when switched to a simple, fully active regimen such as dolutegravir- or CAB-based therapy with LEN that they could adhere to. Therefore, a careful discussion about OBR adherence when starting LEN is critical to avoid functional LEN monotherapy and LEN resistance developing.

The median reduction in pill burden from 2 daily pills to 1 daily pill following LEN initiation may represent a clinically meaningful advance for many HTE PWH, particularly those facing pill fatigue, dysphagia, or gastrointestinal intolerance to oral ART [20]. While the overall number of antiretroviral agents remained unchanged, the simplification of daily dosing combined with LEN's long-acting subcutaneous administration likely facilitated adherence and contributed to sustained suppression. ISRs were common but generally tolerated, leading to discontinuation in only 1 PWH. These findings are consistent with previously reported tolerability profiles, suggesting that, with appropriate patient education and anticipatory guidance, LEN's injection-related side effects are manageable in routine clinical care [19].

The demographic and clinical characteristics of this cohort, predominantly Black, publicly insured, and with frequent socioeconomic and structural vulnerabilities, highlight LEN's potential to improve outcomes among populations historically underrepresented in ART trials [21]. Several PWH initiated LEN not solely for resistance, but also to address barriers such as poor oral medication tolerance, unstable housing, and complex adherence needs, with people with non-MDR HIV experiencing the same frequency of viremia resolution as those with MDR HIV. These pragmatic applications illustrate LEN's utility beyond the narrow confines of “salvage therapy,” extending its relevance to broader HIV treatment equity and retention efforts.

However, implementation of LEN at scale will require deliberate system-level strategies. The medication's success is closely tied to OBR adherence and consistent clinic attendance for twice-yearly injections. Equitable access will also depend on navigating payer coverage, prior authorization requirements, and integration into public health programs such as Medicaid, Medication Assistance Program/AIDS Drug Assistance Program, and Ryan White [22]. In addition, the absence of commercially available genotypic assays capable of detecting LEN-associated resistance poses an emerging challenge [23]. This is of particular importance as LEN is also used as HIV PrEP and may therefore not be an option for treatment if someone develops HIV infection via breakthrough with optimal adherence or during the long pharmacokinetic tail of LEN activity after stopping LEN injections and not receiving additional PrEP [24]. Routine access to such testing would be particularly valuable in cases of incomplete suppression or suspected treatment failure, as observed in a small number of individuals in this cohort, and will be essential as LEN use expands.

This study has several important limitations. The retrospective design limits causal inference and introduces potential confounding by unmeasured variables such as objective measures of OBR adherence, social instability, or comorbid conditions. Data abstraction from different electronic medical records by different researchers may have resulted in inconsistent review of records and may impact variables like substance use history, incomplete capture of follow-up VLs, adverse events, or detailed adherence metrics. ISRs may also be underreported, as there was no standardized method for documenting or grading local reactions across participating sites as was done in the clinical trials. Median follow-up of approximately 1 year, while informative for early outcomes, does not capture long-term durability or resistance evolution. Finally, while this cohort included 6 diverse urban clinics, findings may not be generalizable to all settings without dedicated HIV infrastructure or experience in managing complex salvage regimens.

In summary, this multisite, real-world analysis demonstrates that LEN-based regimens yield high rates of virologic suppression, good tolerability, and meaningful regimen simplification among people with and without MDR HIV in a mostly HTE population. LEN represents a major advance in the management of MDR HIV, offering both pharmacologic potency and pragmatic advantages such as reduced pill burden and infrequent dosing. As use expands, targeted implementation strategies focusing on adherence support, OBR optimization, and equitable access will be essential to ensure that LEN achieves its full potential in improving outcomes for those most affected by MDR HIV and structural barriers to care.

## Supplementary Material

ofag425_Supplementary_Data

## Supplementary Data

Supplementary materials are available at *Open Forum Infectious Diseases* online. Consisting of data provided by the authors to benefit the reader, the posted materials are not copyedited and are the sole responsibility of the authors, so questions or comments should be addressed to the corresponding author.
