# Supplementary material for: Virologic Outcomes With Lenacapavir in People With Human Immunodeficiency Virus: A Multicenter Real-World Study
Source: Open Forum Infect Dis. 2026 Jul 9;13(7):ofag425. doi: 10.1093/ofid/ofag425 (PMC13386173; doi:10.1093/ofid/ofag425)
Supplement: ofag425_Supplementary_Data [file ofag425_supplementary_data.docx]

Supplementary Materials:

**Supplemental Table 1: Reverse Transcriptase Mutation Frequency**

| Major Mutations | Frequency N (%) |
| --- | --- |
| M184(V/I) | 51 (75%) |
| K103N | 30 (44%) |
| T215(Y/F/I/N/A/T/D/S/C) | 25 (37%) |
| D67(N/G/H) | 18 (26%) |
| K219(N/Q/E/R) | 18 (26%) |
| K101(E/P/Q) | 16 (24%) |
| K70(R/E/G/Q/N/T) | 16 (24%) |
| T69(D/N/S/T/R) | 16 (24%) |
| M41L | 13 (19%) |
| L210(W/M) | 12 (18%) |
| Y181(C/I) | 12 (18%) |
| V106(I/M) | 10 (15%) |
| Y188(L/H) | 8 (12%) |
| L100I | 7 (10%) |
| K65R | 5 (7%) |
| G190(A/S) | 4 (6%) |
| L74V | 3 (4%) |
| Y115F | 3 (4%) |
| F227(Y/L) | 2 (3%) |
| Q151M | 1 (1%) |
| Minor/Uncertain Significance | |
| E138(A/G/K) | 13 (19%) |
| V179(I/F/L) | 9 (13%) |
| E44(D/K) | 8 (12%) |
| A98(G/S) | 7 (10%) |
| R211(K/Q) | 6 (9%) |
| V118I | 6 (9%) |
| P225H | 4 (6%) |
| F214L | 3 (4%) |
| V108I | 3 (4%) |
| V90I | 3 (4%) |
| Y318F | 3 (4%) |
| A62V | 2 (3%) |
| A71(V/T) | 2 (3%) |
| H208(F/Y) | 2 (3%) |
| I178L | 2 (3%) |
| I93L | 2 (3%) |
| L10I | 2 (3%) |
| L63P | 2 (3%) |
| V77I | 2 (3%) |
| E203D | 1 (1%) |
| G16E | 1 (1%) |
| H221Y | 1 (1%) |
| I50L | 1 (1%) |
| I62V | 1 (1%) |
| I64L | 1 (1%) |
| K104N | 1 (1%) |
| K20(R/T) | 1 (1%) |
| L33F | 1 (1%) |
| L89M | 1 (1%) |
| M36I | 1 (1%) |
| N348I | 1 (1%) |
| Q207E | 1 (1%) |
| R211Q | 1 (1%) |
| S68S | 1 (1%) |
| V189I | 1 (1%) |
| V245T | 1 (1%) |
| V75I | 1 (1%) |
| V75M | 1 (1%) |
| V82I | 1 (1%) |

Data are n (%), assessing the 68 subjects with available genotype data.

This table includes both Nucleoside Reverse Transcriptase Inhibitor and Non-Nucleoside Reverse Transcriptase Inhibitor mutations.

**Supplemental Table 2: Protease Inhibitor Mutation Frequency**

| Major Mutations | Frequency N (%) |
| --- | --- |
| I54(V/L/I/A) | 15 (22%) |
| M46(I/L) | 14 (21%) |
| V82(A/T) | 11 (16%) |
| I84V | 8 (12%) |
| V32I | 4 (6%) |
| I47V | 3 (4%) |
| I50(V/L) | 3 (4%) |
| G48V | 2 (3%) |
| L90M | 2 (3%) |
| N88(S/D) | 2 (3%) |
| L76V | 1 (1%) |
| Minor/Uncertain Significance |  |
| L63(P/A/T/S) | 21 (31%) |
| L10(I/V/F) | 18 (26%) |
| A71(V/T/I) | 14 (21%) |
| V77I | 14 (21%) |
| M36I | 13 (19%) |
| L33(F/I) | 9 (13%) |
| I13V | 7 (10%) |
| I62(V/K) | 6 (9%) |
| K20(R/I/M) | 6 (9%) |
| F53(L/Y) | 5 (7%) |
| L24I | 5 (7%) |
| E35D | 4 (6%) |
| I64V | 4 (6%) |
| I93L | 4 (6%) |
| Q58E | 4 (6%) |
| D60E | 2 (3%) |
| I85V | 2 (3%) |
| K43(T/R) | 2 (3%) |
| L89M | 2 (3%) |
| T74S | 2 (3%) |
| D30N | 1 (1%) |
| E34Q | 1 (1%) |
| G16E | 1 (1%) |
| H69Q | 1 (1%) |
| I15I | 1 (1%) |
| K55R | 1 (1%) |

Data are n (%), assessing the 68 subjects with available genotype data.

This table includes only Protease Inhibitor mutation data.

**Supplemental Table 3: Integrase Strand Transfer Inhibitor Mutations**

| Major Mutations | Frequency N (%) |
| --- | --- |
| E138(K/A/D/T) | 12 (18%) |
| G140(S/A/C) | 10 (15%) |
| Q148(H/R) | 9 (13%) |
| R263K | 8 (12%) |
| N155H | 7 (10%) |
| E92Q | 3 (4%) |
| G118R | 2 (3%) |
| T66I | 1 (1%) |
| Minor/Uncertain Significance |  |
| T97(A/V) | 8 (12%) |
| G163E/R/Q/K | 7 (10%) |
| M50(I/V) | 7 (10%) |
| S230N | 5 (7%) |
| E157(Q/E) | 4 (6%) |
| L74(I/M) | 4 (6%) |
| S147G | 3 (4%) |
| G193(D/E) | 2 (3%) |
| M36I | 2 (3%) |
| V151I | 2 (3%) |
| A71T | 1 (1%) |
| D232N | 1 (1%) |
| F121Y | 1 (1%) |
| H51Y | 1 (1%) |
| H69Q | 1 (1%) |
| L63P | 1 (1%) |
| S153A | 1 (1%) |

Data are n (%), assessing the 68 subjects with available genotype data.

This table includes only Integrase Strand Transfer Inhibitor mutation data.

**Supplemental Table 4: Number of ART Agents**

|  | **Overall** | **MDR HIV** | **Non-MDR HIV** | **p-value** |
| --- | --- | --- | --- | --- |
| N | 70 | 18 | 52 |  |
| Number of Agents in Pre-LEN regimen, median (IQR) | 4 (3-5) | 4 (3-5) | 4 (3-5) | 0.76 |
| 0 | 1 (1%) | 0 (0%) | 1 (2%) |  |
| 1 | 1 (1%) | 0 (0%) | 1 (2%) |  |
| 2 | 7 (10%) | 1 (6%) | 6 (12%) |  |
| 3 | 21 (30%) | 7 (39%) | 14 (27%) |  |
| 4 | 19 (27%) | 3 (17%) | 16 (31%) |  |
| 5 | 16 (23%) | 5 (28%) | 11 (21%) |  |
| 6 | 5 (7%) | 2 (11%) | 3 (6%) |  |
| Number of Agents in Post-LEN regimen, median (IQR) | 4 (3-5) | 4 (3-5) | 3 (2.5-4) | 0.455 |
| 2 | 15 (21%) | 2 (11%) | 13 (25%) |  |
| 3 | 19 (27%) | 5 (28%) | 14 (27%) |  |
| 4 | 18 (26%) | 4 (22%) | 14 (27%) |  |
| 5 | 10 (14%) | 4 (22%) | 6 (12%) |  |
| 6 | 6 (9%) | 3 (17%) | 3 (6%) |  |
| 7 | 2 (3%) | 0 (0%) | 2 (4%) |  |

Data are n (%) or median (IQR).

ART=Antiretroviral Therapy. MDR=Multi-drug Resistant. LEN=Lenacapavir.

**Supplemental Table 5: Pill Burden**

|  | **Overall** | **MDR HIV** | **Non-MDR HIV** | **p-value** |
| --- | --- | --- | --- | --- |
|  | 70 | 18 | 52 |  |
| Number of Pills daily in Pre-LEN regimen, median (IQR) | 2 (1-2) | 2 (1-3) | 1 (1-2) | 0.018 |
| 0 | 6 (9%) | 0 (0%) | 6 (12%) |  |
| 1 | 24 (34%) | 2 (11%) | 22 (42%) |  |
| 2 | 23 (33%) | 9 (50%) | 14 (27%) |  |
| 3 | 10 (14%) | 3 (17%) | 7 (13%) |  |
| 4 | 4 (6%) | 2 (11%) | 2 (4%) |  |
| 5 | 2 (3%) | 1 (6%) | 1 (2%) |  |
| 6 | 1 (1%) | 1 (6%) | 0 (0%) |  |
| Number of Pills daily in Post-LEN regimen, median (IQR) | 1 (0-2) | 1 (1-2) | 1 (0-2) | 0.001 |
| 0 | 21 (30%) | 0 (0%) | 21 (40%) |  |
| 1 | 28 (40%) | 11 (61%) | 17 (33%) |  |
| 2 | 16 (23%) | 4 (22%) | 12 (23%) |  |
| 3 | 3 (4%) | 2 (11%) | 1 (2%) |  |
| 4 | 1 (1%) | 1 (6%) | 0 (0%) |  |
| 5 | 1 (1%) | 0 (0%) | 1 (2%) |  |

Data are n (%) or median (IQR).

MDR=Multi-drug Resistant. LEN=Lenacapavir.
